# On Insanity; Its Nature, Causes, and Cure

**Published:** 1837-01

**Authors:** 


### Art. XIV.

*On Insanity; its Nature, Causes, and Cure.* By Wm. B. Neville, Esq., of Earl's Court House.—*London*, 1836. 8vo. pp. 192.

Attached to this publication is a Prospectus of Mrs. Bradbury's "Establishment" at Old Brompton, for the recovery of *ladies* labouring under affections of the mind. Prefixed to the Prospectus is the appropriate motto, "*Endeavour to be first in thy calling,*" &c. &c.; and Mrs.

Bradbury addresses herself very seriously to the "friends of the nervously affected," assuring them how much is done at Old Brompton "to enhance a healthy progress of the mind in the valetudinarian," in an establishment which she says induces "an impression of gratification in even the most fastidious." Mrs. Bradbury states that, in addition to Mr. Neville, she has introduced "every adaptation bearing upon the curative feature." Of course, we are reminded that "Old Brompton has been *proverbially* styled by the profession, for many centuries, the Montpelier of England." Then we have medical certificates from numerous fashionable physicians; and following upon these we have a ground plan, and after the ground plan seven lithographic pictures, all displaying the beauties of Brompton, and illustrating the doings at its superlative "Establishment."

Thus garnished, the intention of Mr. Neville's publication is sufficiently evident. It is an elaborate advertisement, prepared to order, and only too well executed, we fear, to have satisfied the taste of the excellent head of the ladies' "establishment." We can assure Mr. Neville that we have in our time refused tempting offers to write for such establishments; but we considered that our talents were, as his own assuredly are, worthy of a better engagement.

In short, Mr. Neville has written a very respectable exercise on madness, which, had it been published alone, might have excited surprise, seeing that it was a little uncalled for, but which comes forth bedizened with a portentous *tail* that disfigures and degrades it. It must have been with a prospective regard to this appendage that Mr. Neville laboured, in his introductory pages, to be learned as to the Jews, and the priestesses of Greece, and the "work" of Aretæus, *De Melancholia*, and the "treatise" of Celsus on the same disease, and of Ælius Aurelianus and Alexander Trallianus; by whose names we are forcibly reminded of Sanconiathon, Manetho, and Berosus, as quoted by the learned cosmogonist, Mr. Jenkinson.

The whole plan of Mr. Neville's book is so eclectic, he picks up opinions on every side with such eagerness, and strings them together so oddly, that we can with difficulty gather what his own opinions are, or whether he has any opinions of his own. He agrees with the phrenological pathologists in ascribing insanity to disorder of some of the faculties admitted by them; but he also agrees with M. Foville, that it occurs in persons who have "the most regular and harmonious configuration of the head imaginable." He maintains the old prejudice that poets are particularly prone to madness, and, forgetting Shakspeare, Milton, Pope, Goldsmith, and Thomson, and Southey, and Wordsworth, refers to the absurd story of Marcus, a citizen of Syracuse, who, "when he had lost his senses, became a fine poet;" and adduces also the instance of poor Nat. Lee. He asserts that "our sweet poet Burns was frequently influenced by *delusions* of the imagination;" an assertion which we believe to be altogether groundless. He declares that Mr. Hogg was mad; that Pascal was hypochondriacal; that "the great novelist and poet, Sir Walter Scott, suffered through the same cause;" and that Sophocles was really insane.

We need hardly say that all this is equally absurd and unjust. But the often-refuted scandal against Sophocles must be revived, and Burns and Scott must be declared insane, in order to reconcile "the friends of the nervously affected" to insanity, by persuading them into the weak and baseless opinion that the soundest minds are the most prone to unsoundness.

We turn from page to page of the work with the stronger conviction that, although containing much information collected from various authorities, it is still but a work *got up*, without any intention or design, as it seems to us, except that of interesting the public in Mrs. Bradbury's establishment. All the information it contains has already been before the medical public in better form. The practical chapters might at least be expected to contain valuable matter, the result of experience; and we certainly find them less objectionable and better written than the rest. In what relates to the prognosis, however, to say that puerperal mania "almost always ends favorably and *speedily*" is to run the risk of producing painful disappointment; and we equally object to the doctrine that *mania* occurring in young women at the age of puberty is a disorder as transitory as the delirium of a fever; and also that, in the derangement following habitual indulgence in spirituous liquors, the recovery is generally very speedy.

We think the author is perfectly justified in ascribing the defective treatment of insanity to the neglect of all treatment in the greater number of lunatic establishments. If, as it is stated, the total number of recoveries varies from about thirty to fifty per cent.; if the recoveries at the York Retreat are about eighty-nine per cent. in recent cases, and in others fifty; if the results of Dr. Burrows's experience are the same; and if similar results have been produced by the treatment at the Connecticut Retreat, there is much encouragement to persevere in the application of remedial measure, even although we demur to admitting M. Broussais' confident assertion that he can arrest incipient mania with as much certainty as incipient inflammation; an assertion which Mr. Neville does not seem to doubt.

The recommendation not to neglect attending to the mental and moral and physical state of such idiotic children as do not obviously labour under congenital or organic imperfection in the development of the head, we very highly approve of. Few idiots are totally idiotic. We equally approve of the recommendation not to assume that the brain is incapable of recovery, even after long-continued derangement.

In the treatment of insanity, a favorite remedy with Mr. Neville is the carbonate of soda, either given simply or in infusion of bark, calumba, &c., in doses of one, two, three, and even four drachms at a time. The good effects of this medicine on the general health is illustrated by experiments performed upon two monkeys. Both were treated like rational creatures; that is to say, they were kept in a warm room, took little exercise, and lived extremely well; having tea and bread and butter, and a mutton chop or fried bacon, for breakfast; meat for dinner, with vegetables, tarts, beer, wine, and dessert; in the evening tea, and supper at night, and, as we presume, a glass of brandy and water before going to bed. No favour was shown, but both were treated alike, except that to one of them half a drachm of the carbonate of soda was given three times a day, and to the other not any. This went on for six months; at the end of which time, the one to whom the soda was given was plump and well, whereas the other exhibited every sign of a broken constitution, and was killed for the sake of examination after death. Its muscles were shrunk and pale, the stomach and alimentary canal were congested, the mucous coat of the stomach was thickened, the right lobe of the liver indurated, and the mesenteric glands were much enlarged. The experiment was repeated on two other monkeys, and on two dogs, with the same results. Some *human* cases are added: one of "a gentleman holding a high official situation," and one of a "lady of distinction, whom we" (Mr. Neville,) "visited *for some time*, along with her *ordinary* medical attendant, a *practitioner of eminence*;" but we are content with the cases of the monkeys. The lady of distinction, however, took *an ounce* of the carbonate of soda, in five equal doses, in the course of the four-and-twenty hours, and happily recovered "plumpness and bloom."

We fear Mr. Neville will think we have treated his work with severity. Certainly some passages of it are so sensibly expressed, and some are so miserably affected, that we could imagine he had been tasked with the dressing-up of a common work from another hand; and we need not say that we think his whole performance would have had a better appearance if the Prospectus of Mrs. Bradbury's Establishment and the lithographic ornaments had been omitted.

---

